# Supplementary material for: Morphology and mobility as tools to control and unprecedentedly enhance X-ray sensitivity in organic thin-films
Source: Nat Commun. 2020 May 1;11:2136. doi: 10.1038/s41467-020-15974-7 (PMC7195493; doi:10.1038/s41467-020-15974-7)
Supplement: Supplementary file 1 — Supplementary Information [file 41467_2020_15974_MOESM1_ESM.pdf]

Supplementary Information

Morphology and mobility as tools to control and unprecedentedly enhance X-ray sensitivity in organic thin-films

Inés Temiño and Laura Basiricò et al.

Supplementary Figures

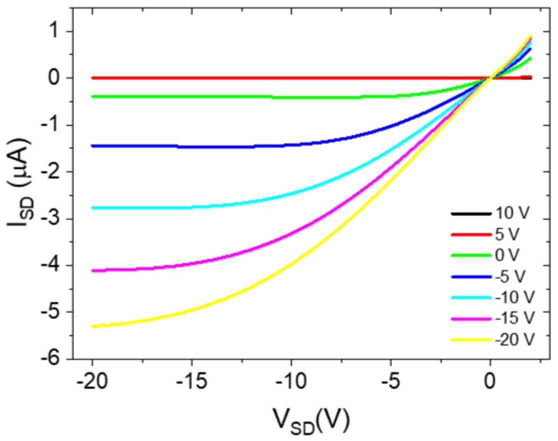

Supplementary Figure 1: **BAMS deposited TIPS-pentacene OFET characteristics.** Typical output characteristics of a TIPS-pentacene OFET.

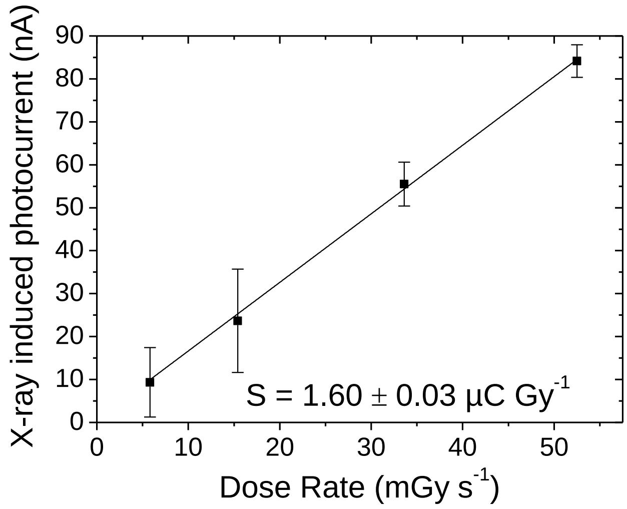

Supplementary Figure 2: **X-ray photocurrent vs. dose rate plot.** X-ray induced photocurrent versus dose rate plot for a TIPS-pentacene device, for which a sensitivity of (1.60 ± 0.03) μC Gy^{-1} is extracted (equivalent to a sensitivity per unit area of $3.8 \cdot 10^{2}$ μC Gy^{-1} cm^{-2}). The error bars refer to the statistical fluctuations (SD) of the signal amplitude over three on/off switching cycles of the X-ray beam in the same condition.

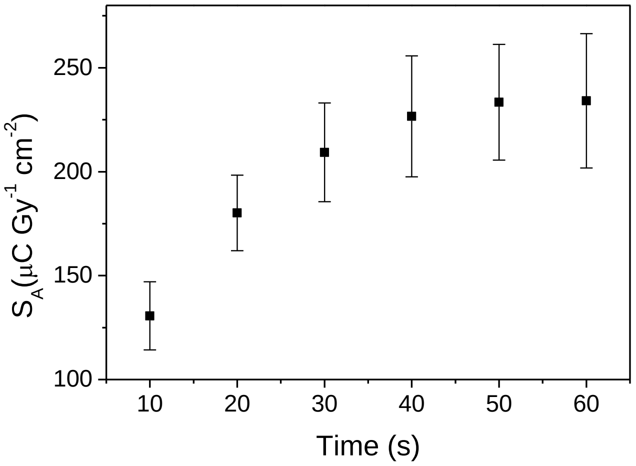

Supplementary Figure 3: Sensitivity variation with time exposure. Sensitivity values over time exposure to X-rays for pure TIPS-pentacene sample. The error bars refer to the fit error over three experimental points (corresponding to three different dose rates), employed for the calculation of the sensitivity values.

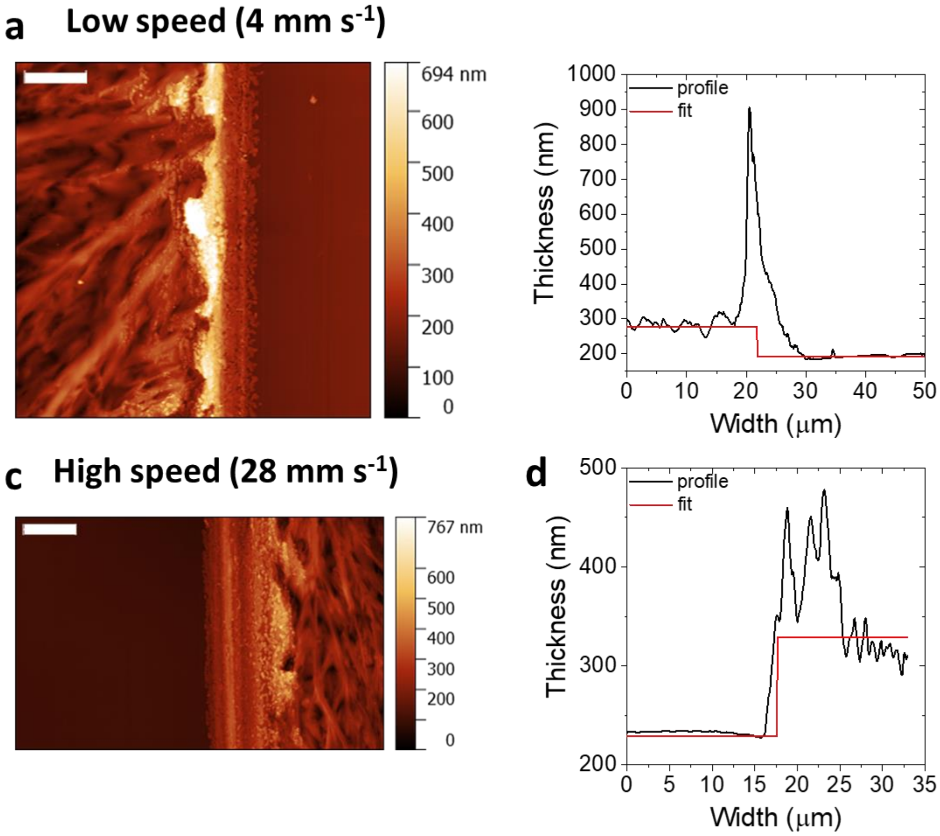

Supplementary Figure 4: **Films' thickness for different deposition speed.** Topographic AFM images (left) and depth profiles (right) for thickness estimation of the TIPS-pentacene:PS thin films deposited at (a) low speed and (b) high speed. Scale bar: 5 μm.

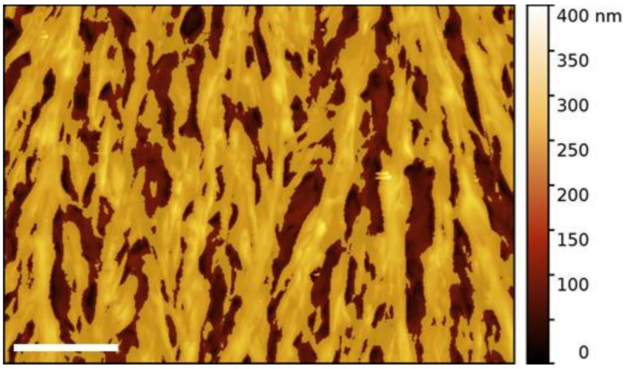

Supplementary Figure 5: Determination of grain size. Gwyddion tool dedicated to the grain size analysis. The yellow areas are different grains of the same semiconducting thin film. Scale bar: 10 µm.

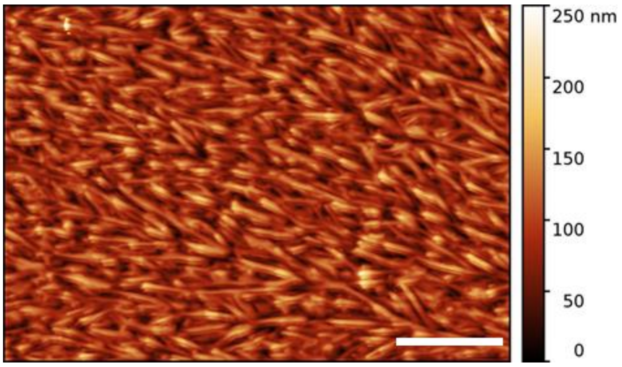

Supplementary Figure 6: **AFM characterization.** AFM topography image of a TIPS-pentacene film deposited at 10 mm s^{-1} (standard speed). Scale bar: 10 µm.

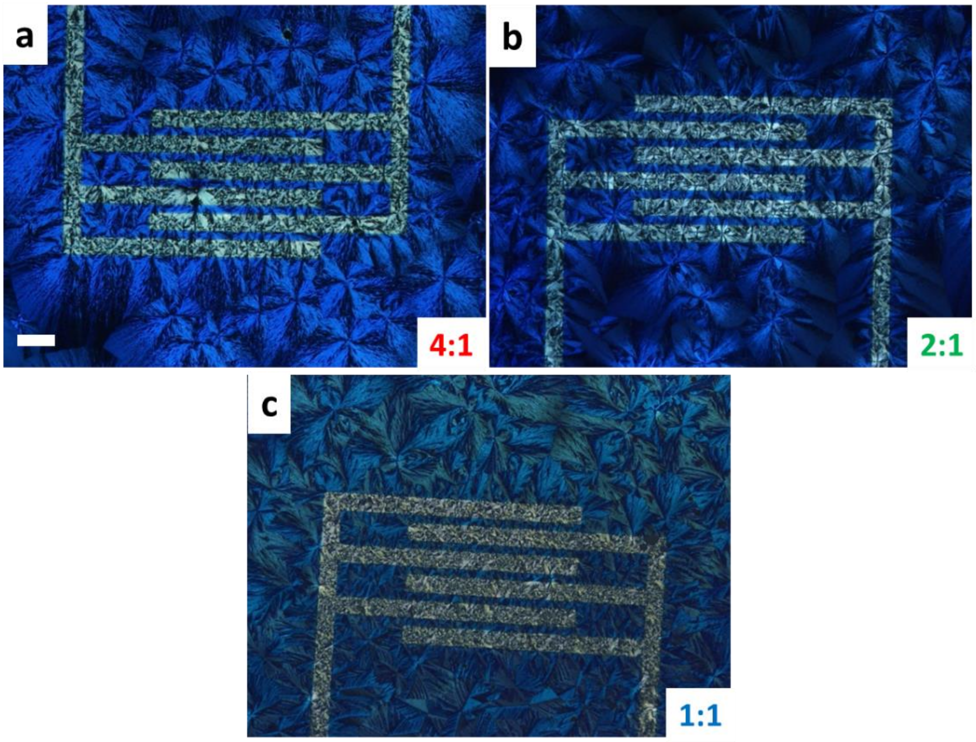

Supplementary Figure 7: **Spherulitic morphology of TIPS-pentacene:PS samples.** Cross-polarised optical microscope images of TIPS-pentacene:PS thin films with different blending ratios (4:1, 2:1, 1:1). Scale bar: 100 µm.

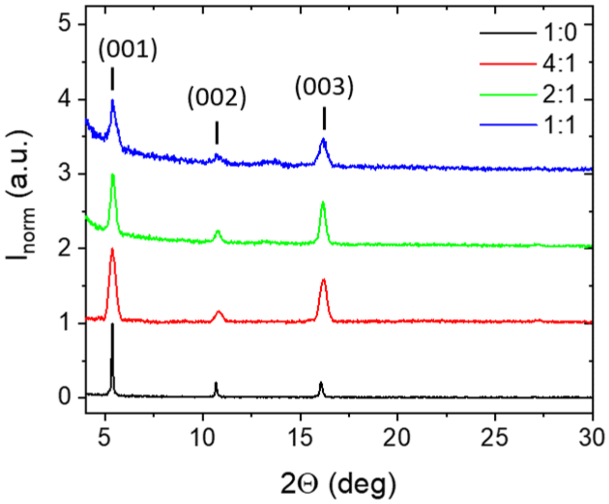

Supplementary Figure 8: **X-ray diffraction measurements.** Normalised X-ray diffractograms of the 1:0, 4:1, 2:1 and 1:1 TIPS-pentacene:PS thin films.

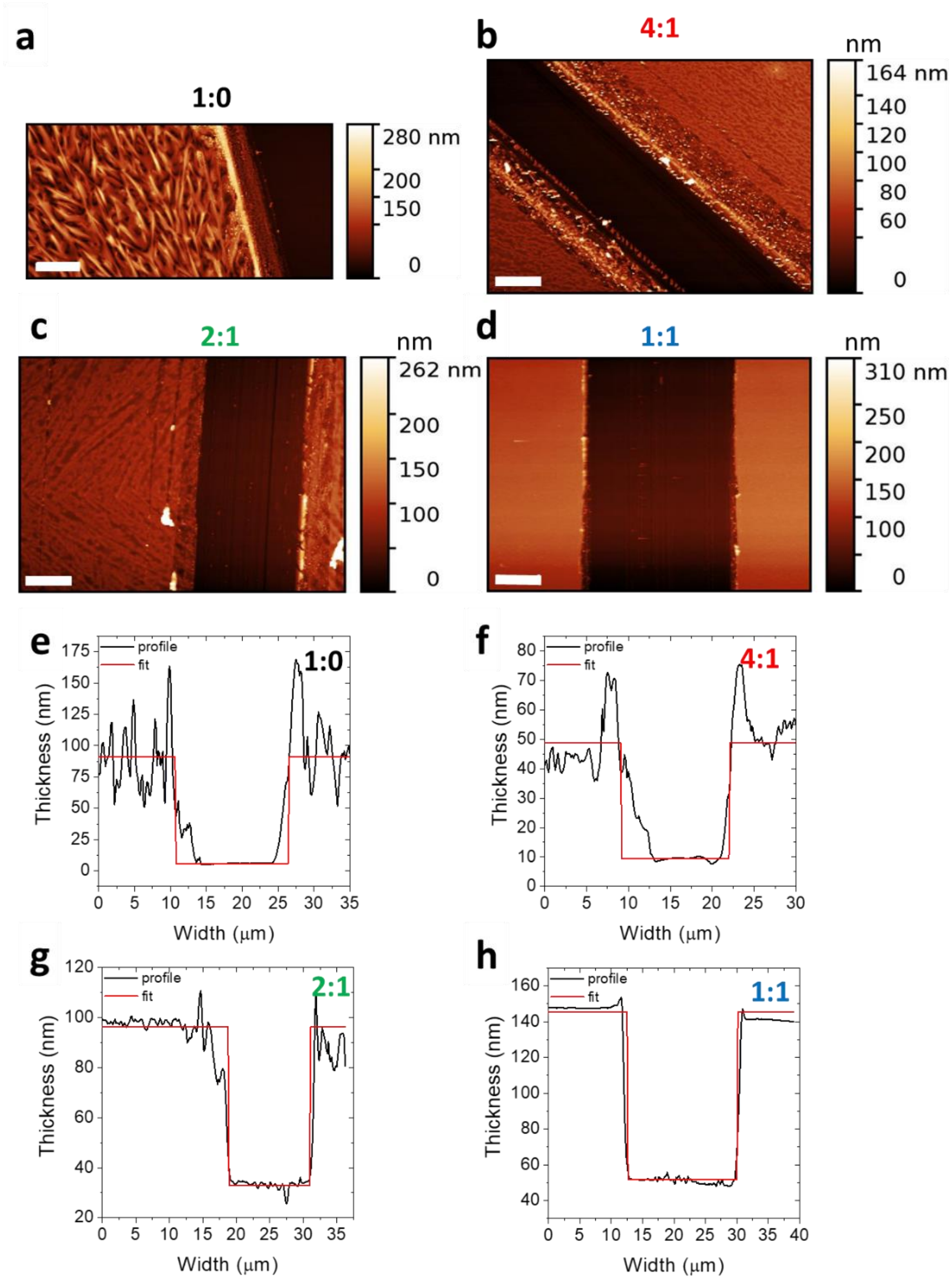

Supplementary Figure 9: **Thickness measurements for films with different blend ratios**. (a) Topographic AFM images and (b) depth profiles used for thickness estimation of the 1:0, 4:1, 2:1 and 1:1 TIPS-pentacene:PS thin films. Scale bar: 5 μm.

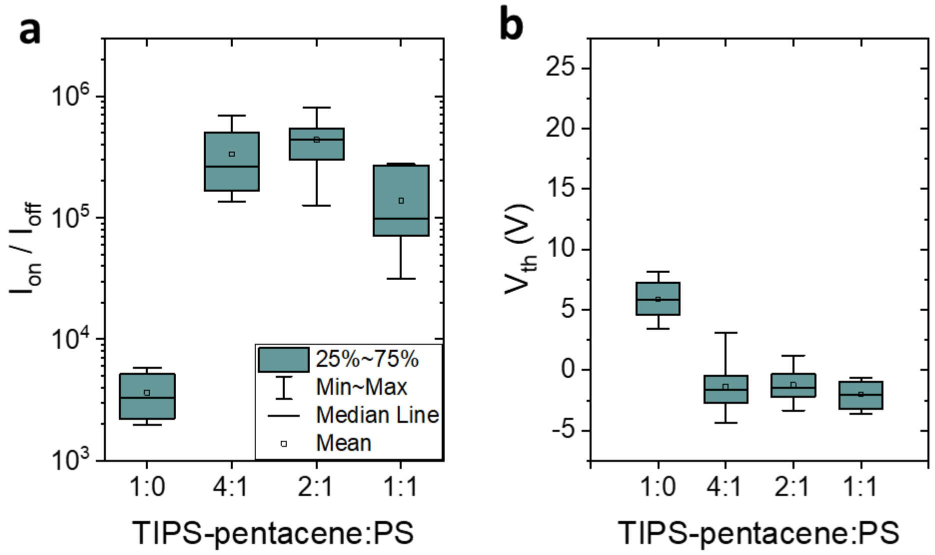

Supplementary Figure 10: **Analysis of the blend ratio effect on on/off ration and threshold voltage.** Box-plot statistics for (a) on/off ratio and (b) threshold voltage of OFET devices based on 1:0, 4:1, 2:1 and 1:1 TIPS-pentacene:PS blends (. The error bars refer to the statistical fluctuations (SD) of the parameters over 12 samples for each blend ratio.

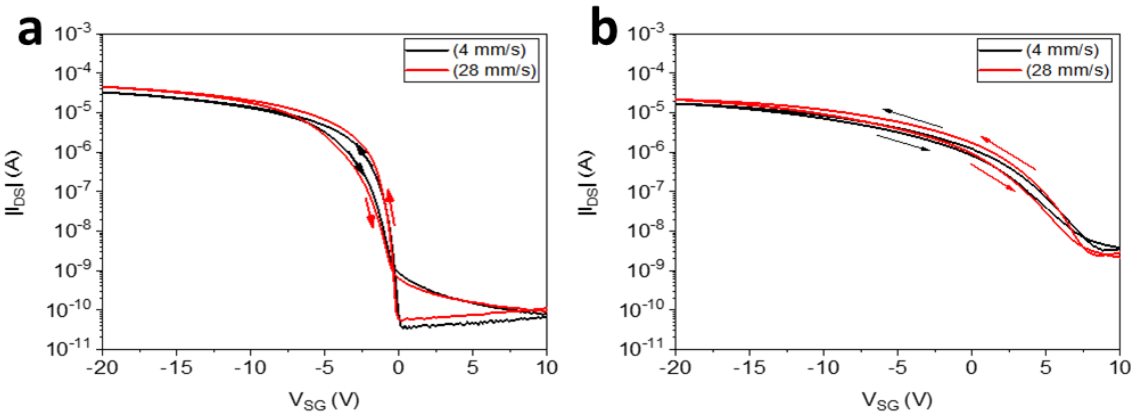

Supplementary Figure 11: **Reduction of hysteresis with deposition speed.** Transfer characteristics of (a) TIPS-pentacene:PS and (b) bare TIPS-pentacene 1:0 OFETs for low (4 mm s^{-1}) and high (28 mm s^{-1}) deposition speed.

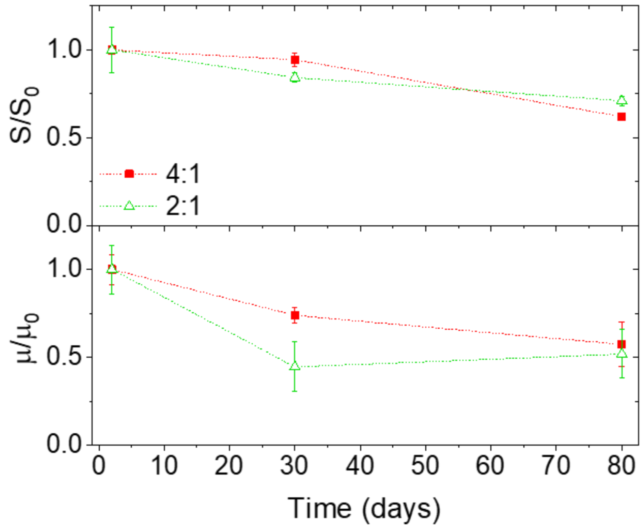

Supplementary Figure 12: Ageing effect on mobility and sensitivity in blended detectors. Evolution over time up to 80 days of X-ray sensitivity (up) and field-effect mobility (down) of 4:1 and 2:1 TIPS pentacene:PS devices. It is noteworthy that the here reported devices were not encapsulated. Values have been normalised to the initial values. The error bars refer to the statistical fluctuations of the parameters over 8 samples.

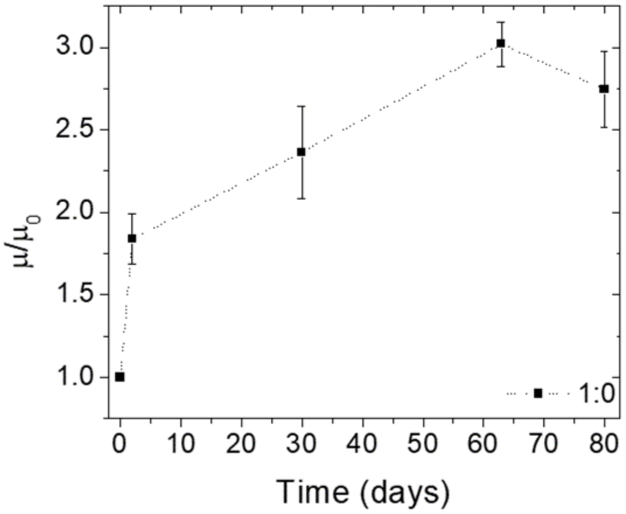

Supplementary Figure 13: **Ageing effect on mobility and sensitivity in pure TIPS-pentacene detectors.** Evolution over time up to 80 days of field-effect mobility of 1:0 TIPS-pentacene:PS devices. Values have been normalised to the initial values. The error bars refer to the statistical fluctuations (SD) of the parameters over 8 samples.

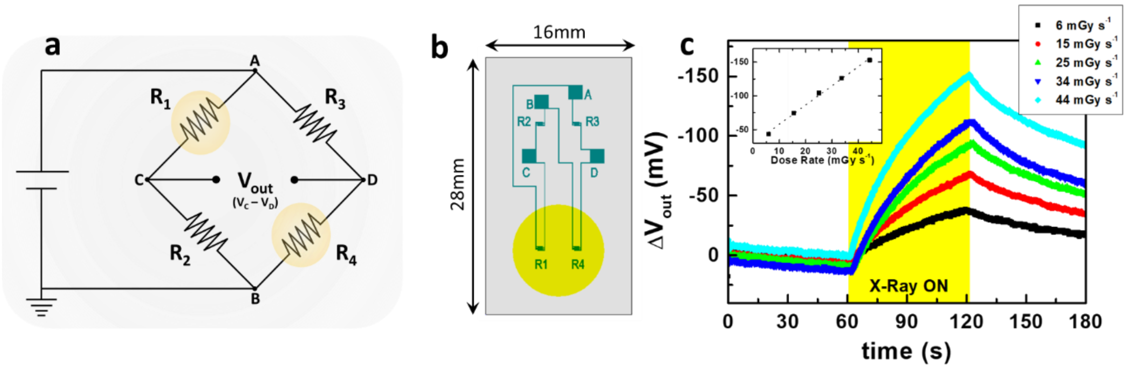

Supplementary Figure 14: Wheatstone bridge-based X-ray detector (a) Wheatstone bridge circuit and (b) schematic representation of the layout designed for the fabrication of the devices. The resistors R_1, R_2, R_3 and R_4 consist in the channel of four different transistors based on 4:1 TIPS-pentacene:PS. During the exposure to X-rays, only R_1 and R_4 are irradiated while R_2 and R_3 are kept in dark. Because of the decrease of the channel resistance due to the photocharges generated by the radiation the unbalanced bridge causes a ΔV_{out} between the C and D nodes. (c) ΔV_{out} generated by different intensity of the X-ray beam and applying V_{SG} = -5 V at the gate contacts of the OFETs and -20 V to the entire circuit. In the inset it is reported the linear response of the circuit for different doses of radiation.

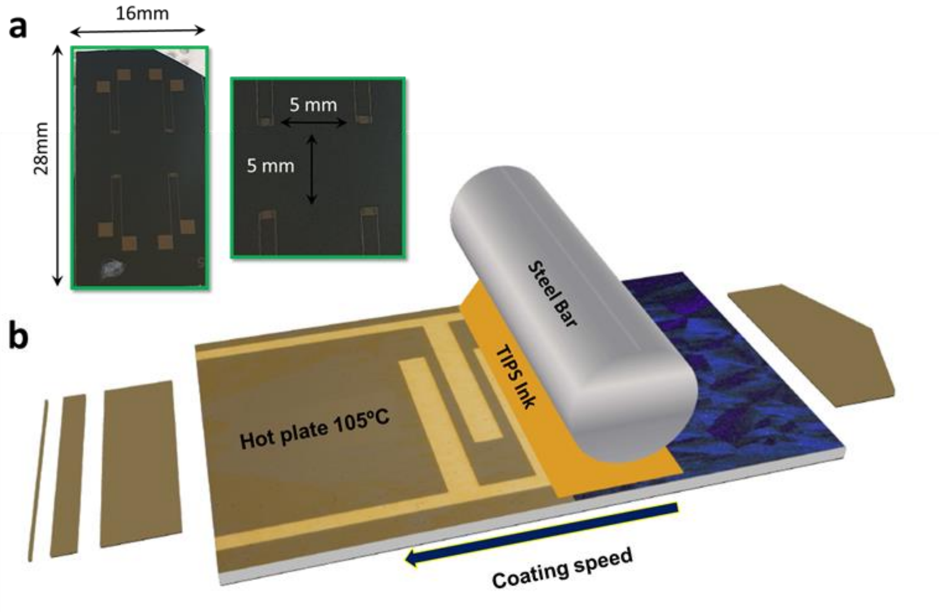

Supplementary Figure 15: **BAMS technique.** Pictures of the samples indicating the substrate dimensions and the spacing between the devices (a). Sketch of the home-built BAMS setup for the deposition of the organic semiconductor (b).

Supplementary Tables

Supplementary Table 1: **OFET parameters for different ratio of TIPS-pentacene:PS blends.**

Blend ratio	I_{on}/I_{off}	Mobility ($cm^2\ V^{-1}\ s^{-1}$)	V_{th} (V)	SS ($V\ dec^{-1}$)	N_T ($10^{12}\ eV^{-1}\ cm^{-2}$)
**1:0**	$(4 \pm 2) \cdot 10^3$	$(1.0 \pm 0.3) \cdot 10^{-2}$	6 ± 2	1.6 ± 0.4	2.7 ± 0.7
**4:1**	$(3 \pm 2) \cdot 10^5$	$(5 \pm 2) \cdot 10^{-1}$	-1 ± 2	0.6 ± 0.2	1.0 ± 0.3
**2:1**	$(4 \pm 2) \cdot 10^5$	$(5 \pm 3) \cdot 10^{-1}$	-1 ± 1	0.5 ± 0.1	0.8 ± 0.1
**1:1**	$(1.4 \pm 0.9) \cdot 10^5$	$(6 \pm 4) \cdot 10^{-2}$	-2 ± 1	0.7 ± 0.1	1.2 ± 0.2

Supplementary Table 2: **Roughness (*rms*) and thin-film thickness for different blend ratios.**

Blend ratio	*rms* (nm)	Thickness (nm)
**1:0**	37	80 ± 20
**4:1**	6	40 ± 10
**2:1**	9	60 ± 5
**1:1**	2	90 ± 5

Supplementary Notes

Supplementary Note 1: Towards large-area X-ray sensors, Wheatstone bridge architecture

The Wheatstone bridge is a widely used device architecture which allows to measure an unknown resistance value and it is typically used in resistive sensor systems. In fact, as illustrated in **Supplementary Figure 15a**, when the bridge is balanced (i.e. the four resistances are equal) the current flowing in the two branches is equal and the voltage difference between the C and D nodes is $V_{out} = 0$ V. If the bridge is unbalanced (i.e. the four resistances are not equal), the electrical currents flowing in these two branches is different and $V_{out} \neq 0$ V. Therefore, by monitoring the voltage difference between C and D it is possible to employ the Wheatstone bridge architecture as a detector sensitive to external stimuli able to modify the resistance of at least one of the resistors of the system. As the Wheatstone bridge architecture is very sensitive to small differences in any one of the four resistances, it is crucial to employ a processing technique for large-area deposition that grants a high degree of film uniformity.

In this work, thanks to the excellent and uniform properties of the organic devices fabricated with the BAMS technique, we realised a Wheatstone bridge consisting of four 4:1 TIPS-pentacene:PS OFETs that operate as resistors, with the layout reported in **Supplementary Figure 15b.** In fact, when X-ray photons impact onto the OFETs, the OFET channel resistance decreases due to the photocurrent increase, as previously discussed. We thus turned the bridge state from the balanced to the unbalanced state by exposing to X-rays only two OFETs, R_1 and R_4 in the reported case. In **Supplementary Figure 15c** the voltage difference between the C and D nodes generated by different dose rates of X-ray radiation is reported. As it can be observed in the inset of the figure, the Wheatstone bridge response to increasing dose rates is linear, as expected in this range of dose rates (see **Fig. 2e**).[1]

Supplementary References

1. Basiricò, L. *et al.* Direct X-ray photoconversion in flexible organic thin film devices operated below 1 V. *Nat. Commun.* **7**, 13063 (2016).
